# Optimized Protocol for Isolation and Culture of Primary Human Corneal Epithelial Cells

**DOI:** 10.1167/tvst.14.10.28

**Published:** 2025-10-22

**Authors:** Rongshan Yan, Feeling Y. Chen, Ethan S. Lindgren, Qi Gao, Yien-Ming Kuo, Danielle M. Robertson, Onur Cil, Matilda F. Chan, Neel D. Pasricha

**Affiliations:** 1 Department of Ophthalmology, University of California San Francisco, San Francisco, CA, USA; 2 Department of Cell & Tissue Biology, University of California San Francisco, San Francisco, CA, USA; 3 Department of Cardiovascular Disease, The first Hospital of Jilin University, Changchun, China; 4 Department of Pediatrics, University of California San Francisco, San Francisco, CA, USA; 5 Department of Ophthalmology, UT Southwestern Medical Center, Dallas, TX, USA

**Keywords:** primary human corneal epithelial cells, cell culture, cell differentiation, cell proliferation, calcium

## Abstract

**Purpose:**

To establish a reliable method for isolating and culturing high-purity primary human corneal epithelial cells (HCECs) for ophthalmic drug testing.

**Methods:**

We present a detailed, step-by-step protocol for the efficient isolation and culture of primary HCECs. This protocol includes the characterization of HCEC morphological responses to varying Ca^2+^ concentrations in the culture medium. Additionally, immunofluorescence staining with well-established markers is used to identify and confirm the cell types in vitro. A Ca^2+^ assay is performed to validate the functionality of the cultured primary HCECs.

**Results:**

By following the procedures detailed in this protocol, high-purity primary HCECs with strong proliferative capacity and preserved morphological integrity are obtained. Immunofluorescence staining confirms the presence of both limbal stem cells and differentiated corneal epithelial cells in vitro. Additionally, the functional assay demonstrates that the cultured primary HCECs retain the ability to respond to external Ca²⁺ stimuli.

**Conclusions:**

This optimized protocol enhances the efficiency and reliability of primary HCECs isolation and culture, enabling the development of a robust in vitro model for studying the mechanisms of ocular diseases.

**Translational Relevance:**

Successfully cultured primary HCECs in vitro bridges the gap between laboratory findings and clinical applications, facilitating advancements in therapeutic development.

## Introduction

The corneal epithelium, which directly interfaces with the external environment, is a primary target for therapeutic eye drops used to treat ocular surface diseases.^1^^,^^2^ Evaluating drug efficacy requires preclinical models that accurately replicate corneal epithelial function. The immortalized human corneal epithelial cell line (HCECs), such as the HCE-2 [50.B1] line immortalized using an adenovirus 12-SV40 virus hybrid (ATCC, CRL-3582), has been widely used as a model to study the corneal surface.^3^ However, these immortalized cells exhibit altered gene expression profiles compared to primary cells and require prolonged air-liquid interface cultures (>1 month) to achieve physiological protein expression, leading to inconsistent results.^4^^,^^5^ A telomerase-immortalized HCEC line (hTCEpi) was found to have characteristic corneal gene expression and phenotypic markers, making it more suitable for studying the mechanisms regulating corneal epithelial cell differentiation.^5^ Compared to immortalized cells, primary HCECs directly derived from donor corneoscleral buttons provide a more physiologically relevant platform for therapeutic drugs development. However, successfully culturing primary cells in vitro has challenges such as low purity, variable yield, limited passages, and slow proliferation. Additionally, conventional serum-free medium prioritizes HCECs’ expansion instead of differentiation. Finally, there is a lack of standardized culture protocols to optimize for functionality, proliferation, and passage potential.

This protocol provides a standardized method for isolating, culturing, and validating primary HCECs, effectively addressing existing challenges. By adapting a mouse corneal epithelial cell culture technique^6^ and using a specialized surgical device for epithelial dissection, we achieved a high yield of primary HCECs that could be subcultured for up to five passages. These cultured primary HCECs exhibit a rapid growth rate, and their functionality was confirmed by a Ca^2+^ assay showing intracellular Ca^2+^ release to ATP stimulation. In summary, our study effectively addresses key challenges in primary cell culture, including cell yield, purity, and functional consistency in primary cell culture. We demonstrate that primary HCECs cultured using the detailed approaches outlined here serve as robust, physiologically relevant tools for modeling ocular surface diseases in vitro.

## Methods

Fresh human corneoscleral buttons with no corneal epithelial defect or opacities were provided by Sierra Donor Services Eye Bank (West Sacramento, CA, USA). These tissues were obtained from deidentified donors with no history of visual or ocular disease (age 18–70 years) and death-to-preservation time less than 24 hours. Donor selection was not biased based on race or ethnicity. The human tissue experiments complied with the guidelines of the ARVO Best Practices for Using Human Eye Tissue in Research.

### Solution Preparation

#### Culturing Medium

##### Complete Growth Medium

1) The complete primary HCECs growth medium contains serum-free Corneal Epithelial Cell Basal Medium (ATCC, PCS-700-030) and is supplemented with the Corneal Epithelial Cell Growth Kit (ATCC, PCS-700-040, Table 1).
2) The Ca^2+^ concentration in the commercial Corneal Epithelial Cell Basal Medium is 0.0612 mM. No Ca^2+^ is present in the commercial Corneal Epithelial Cell Growth Kit.
3) Add 1%–2% Penicillin/Streptomycin solution (P/S solution, Supplementary Table of Materials) into the complete growth medium to avoid contamination during cell culture.

##### Differentiation Medium

1) Dilute the 1 M CaCl₂ stock solution with complete growth medium to prepare differentiation media containing either 0.11 mM or 1.06 mM Ca^2+^.
2) The low Ca^2+^ group contains 0.11 mM Ca^2+^, composed of 0.06 mM from the complete growth medium plus 0.05 mM additional Ca^2+^ supplementation.
3) The normal Ca^2+^ group contains 1.06 mM Ca^2+^, composed of 0.06 mM from the complete growth medium plus 1 mM additional Ca^2+^ supplementation.

##### Subculture Reagents

1) The 0.05% Trypsin-0.02% ethylenediamine tetra-acetic acid (EDTA) (Supplementary Table of Materials) to detach the primary HCECs from the cell culture flasks bottom.
2) Trypsin-neutralizing solution (PBS without Ca^2+^ and Mg^2+^, supplemented with 5% fetal bovine serum solution, Supplementary Table of Materials) to stop the trypsin reaction.

#### Corneoscleral Buttons Preservation Reagents

Corneoscleral buttons are preserved in Optisol GS corneal storage media, which contains dextran, 2.5% chondroitin sulfate, vitamins, precursors of ATP (adenosine, inosine, and adenine), gentamicin, and streptomycin.

#### Corneoscleral Buttons Digestion Solution

1) Prepare digestion solution the same day receiving the corneoscleral buttons. The digestion solution consists of Dispase II enzyme (Supplementary Table of Materials) and D-sorbitol^7^ (Supplementary Table of Materials).
2) Dissolve Dispase II enzyme in cold complete growth medium to a final concentration of 15 mg/mL.
3) Add an appropriate volume of D-sorbitol stock solution (1 M) to achieve a working concentration of 100 mM.
4) We recommend using 5 mL complete growth medium for digesting one corneoscleral button, then 500 µL D-sorbitol stock solution to make the digestion solution.

### Supplies

See Supplementary Table of Materials.

### Primary HCECs Isolation and Culturing Procedures

All procedures are conducted inside the biosafety cabinet with continuous ventilation. Before starting the experiments, the UV light is turned on for at least 15 minutes. After completing the experiments, the biosafety cabinet is decontaminated by spraying it thoroughly with 70% ethanol. Ten total human corneoscleral buttons were used in this study. Each study comparing various Ca^2+^ concentrations used HCECs from the same donor to avoid inter-donor variability.

#### Primary HCECs Isolation

##### Corneoscleral Button Dissection

1) Corneoscleral button storage: Human donor corneoscleral buttons are preserved in the preservation reagent (see **Step 1.2.** for recipe) and maintained at 2°C to 8°C until further processing.
2) Rinsing procedures:
  • Using forceps, carefully transfer a single corneoscleral button into a 60 mm × 15 mm petri-dish.
  • Rinse the corneoscleral button three times with cold 1% P/S containing Hanks’ balanced salt solution (HBSS; Supplementary Table of Materials). Each corneoscleral button should be processed individually in a separate petri-dish or centrifuge tube. Avoid cross-contamination by ensuring no shared tools are used during subsequent steps.
3) Scleral removal: Using a surgical scissor (Supplementary Table of Materials), carefully trim away any remaining scleral tissue to prevent contamination from non-corneal epithelial cells (see Supplementary Video S1).

##### Corneoscleral Button Digestion

1) Prepare the digestion solution as outlined in Step **1.3**. Ensure all steps are performed on ice to preserve enzyme activity.
2) The entire corneoscleral button, including the cornea with intact limbus and scleral rim, is used for epithelial isolation. Each corneoscleral button is placed into a 50 mL conical tube, ensuring it is fully immersed in 5 mL of the digestion solution.
3) Incubate the cornea button at 4°C for at least 16 hours but no more than 24 hours to complete the digestion process.

#### Coating Procedures

The extracellular matrix provides essential structural and biochemical support to primary HCECs. However, when isolated from tissue, corneal epithelial cells rapidly lose their native microenvironment, leading to morphological and functional alterations.^8^^–^^13^ Matrigel is derived from Engelbreth-Holm-Swarm mouse tumor producing large quantities of extracellular matrix proteins and serves as a basement membrane.^14^ Matrigel has been used before for culturing primary mouse corneal epithelial cells.^6^ To select the best coating substrates for culturing primary HCECs, we tested several common coating solutions like laminin, collagen II, and fibronectin in addition to Matrigel. However, only Matrigel-coated flasks or plates consistently supported the healthy growth and maintenance of primary HCECs up to passage 6. Notably, alternative coatings often failed to support robust cell attachment and proliferation even from the first passage. Additionally, once primary HCECs were successfully established using Matrigel, subsequent passages (from passage 3 onward) no longer required any additional coating for continued in vitro culture. Thus, to ensure the primary HCECs attachment and survival, a diluted Matrigel matrix solution (unfiltered) is used to pre-coat culture flasks or plates before plating the primary HCECs in vitro.

1) Storage and handling of matrigel matrix solution:
  • Matrigel matrix LDEV-free solution (Supplementary Table of Materials) is supplied as a concentrated liquid. The original bottle should be stored at −20°C for long-term use and aliquots (200 µL per 1.5 mL tube) are thawed at 4°C at least four hours before use.
  • Add 1% penicillin/streptomycin solution (P/S solution; Supplementary Table of Materials) to cold Ca^2+^- and Mg^2+^-free Dulbecco's phosphate-buffered saline solution (D-PBS, Supplementary Table of Materials) to prevent microbial contamination from coating solution.
  • Prepare a 1:30 dilution of the Matrigel matrix in D-PBS containing 1% P/S, and select the volume based on the bottom surface area of the specific flasks or plates used (see Table 2).
2) Coating and incubation:
  • Use freshly coated plates or flasks on the same day as sub-culturing or plating primary HCECs.
  • Add the prepared coating solution gently in a “Z” pattern into the cell culture flasks or plates inside the biosafety cabinet to ensure even distribution and prevent bubble formation. (For even cell distribution in 96-well microplates, gently tap the bottom center of each well and avoid hitting the wall when putting microplates into the incubator.)
  • Incubate the coated flasks or plates at room temperature (RT) in the biosafety cabinet for one hour, and avoid any exposure to UV light.
  • After incubation, remove the diluted Matrigel matrix solution (it can be reused if stored at 4°C). Rinse the coated-flasks or plates three times with RT Ca^2+^- and Mg^2+^-free D-PBS, which contains 1% P/S solution.
3) Cold handling precaution: To avoid Matrigel polymerization into a three-dimensional hydrogel before plating the primary HCECs, maintain the reagent at 4°C through the entire coating and plating process.

#### Harvesting the Primary HCECs

1) Pre-warm the complete growth medium in a 37°C water bath.
2) Transfer the digested corneoscleral buttons into petri dishes containing the complete growth medium (10 mL per petri-dish, one corneoscleral button per petri-dish).
3) Use the Epi-Bowman's Keratectomy (EBK-Clear device, EP-10-007; Orca Surgical Ltd, Phoenixville, PA, USA; Supplementary Table of Materials) to carefully remove the corneal epithelium without traumatizing the underlying stroma and only harvest the epithelial sheet from the anterior corneal surface, ensuring that only corneal epithelial cells are collected for subsequent steps (Supplementary Video S2).
4) This method enables removal of the entire sheet of corneal epithelium from the corneoscleral buttons, providing enough primary HCECs for subsequent in vitro culture (Fig. 2). (Primary HCECs, whether isolated as a whole sheet or as tissue fragments after digestion from corneoscleral buttons, seemed to exhibit comparable viability, proliferation and differentiation in vitro, irrespective of limbal cell content.
5) Use a 10 mL transfer pipette to collect the primary HCECs along with the 10 mL complete growth medium from the petri-dish and transfer into a 15 mL conical tube (primary HCECs from a single cornea should be collected in one tube).
6) Break up the epithelial cell sheet into single-cell suspension by pipetting with a 1 mL pipette at RT until no obvious tissue sheets remain.
7) Spin the cell suspension in a centrifuge at 800*g* for five minutes and aspirate the supernatant using a sterilized tube connected to a vacuum inside of the biosafety cabinet.
8) Discard the supernatant and resuspend the primary HCECs pellet using 5 mL of complete growth medium.
9) Use the sterilized pipette to transfer the suspended primary HCECs from the conical tube into a Matrigel matrix solution pre-coated T25 flask. The isolated primary HCECs from one cornea button should be cultured in the same flask.
10) Shake the flask to evenly distribute HCECs. Plate all harvested passage one primary HCECs into the prepared T25 flasks. There is no specific cell density requirement at this stage.
11) Forty-eight hours after harvesting the first passage of primary HCECs, wash the cultured primary HCECs three times with sterilized D-PBS containing 1% P/S solution. Replace the cultured HCECs with fresh complete growth medium (5–6 mL per T25 flask) to remove unattached cells.

#### Subculturing Primary HCECs

1) Subculture the primary cultured HCECs when the cells reach 70%–90% confluence. Typically, the first passage of primary HCECs reaches 90% confluence within one week, while two to four passages primary HCECs only take 24–48 hours.
2) Change the medium every two days after the first passage of HCECs.
3) Pre-warm the complete growth medium, 0.05% Trypsin-0.02% EDTA solution and Trypsin-neutralizing solution in a 37°C water bath.
4) Aspirate the floating cells and rinse the cells three times with 1% P/S-containing sterilized D-PBS solution.
5) Add 5–6 mL pre-warmed Trypsin-EDTA solution to fully cover the T25 flask bottom. Incubate the flask in a 37°C incubator for one to two minutes to accelerate the cell detachment, if needed.
6) Check the primary HCECs’ morphology under the microscope. If most of the cultured HCECs have formed a spherical shape, they can easily detach from the flask bottom with gentle tapping from sides. Add equal volume of Trypsin-neutralizing solution into the flask. Fully mix the solution and make sure all the trypsin reaction has been stopped in the flask.
7) Transfer the dissociated HCECs along with the 0.05% Trypsin-0.02% EDTA and Trypsin-neutralizing solution mixture into a conical tube, then spin in a centrifuge at 800*g* for five minutes.
8) Aspirate the supernatant and add 5–6 mL of complete growth medium to resuspend the cells.
9) Count the cells, then dilute the HCECs in complete growth medium to a density of 10^6^ cells and plate them into a Matrigel matrix pre-coated T75 cell culture flask or 96-well plate at 30,000 cells per well.
10) Incubate the newly seeded flasks or plates in a humidified incubator at 37°C, 5% CO_2_. Check the HCEC's condition every two days.

### Immunofluorescence Analysis of Primary HCECs

#### Solution Preparation

1) Fixation solution: Dilute the 16% paraformaldehyde (formaldehyde) aqueous solution (PFA solution; Supplementary Table of Materials) to 4% using 1X D-PBS solution.
2) Blocking and Permeabilization Solution: 1× D-PBS solution containing 5% normal donkey serum (NDS) and 0.3% Triton X-100.
3) Antibody Dilution Solution: Primary or secondary antibodies directly diluted in D-PBS solution containing 5% NDS and 0.1% Triton X-100, at certain ratios (Supplementary Table of Materials).

#### Plating the Cells

Primary HCECs from passage 2 and beyond are ideal for immunostaining experiments.

1) Pre-warm the complete growth medium in a 37°C water bath. Prepare the Matrigel matrix coated 96-well, glass bottom plate on the same day (Step 3.2).
2) Follow the cell sub-culturing procedure to digest the cultured HCECs (Step 3.4) and resuspend them in the complete growth medium. Plate the cells at a density of 30,000 cells per well.

#### Staining Procedures

Immunofluorescence staining should begin when the plated primary HCECs reach 90% confluence.

1) Cell washing and fixation:
  • Remove the complete growth medium and thoroughly rinse each well with 250–300 µL PBS three times.
  • Fix primary HCECs with 4% PFA at RT for 20 minutes.
2) Post-fixation wash: Aspirate the fixation solution and rinse the cells once with 200–250 µL PBS, followed by three additional washes with 100 µL PBS.
3) Permeabilization and blocking:
  • Permeabilize the cells using 0.1% TritonX-100 for 20 minutes at RT.
  • Block nonspecific binding by incubating HCECs with 5% NDS for 30 minutes at RT.
4) Primary antibody incubation:
  • Remove blocking solution and incubate primary HCECs with different primary antibodies diluted in the antibody dilution solution overnight at 4°C.
  • The dilution factors for various primary antibodies are described in the Supplementary Table of Materials.
5) Primary antibody wash: Remove the primary antibody dilution solution, rinse the cells thoroughly with 200–250 µL PBS per well, and wash them with 100 µL PBS three times at RT.
6) Secondary antibody incubation:
  • Dilute all secondary antibodies (Supplementary Table of Materials) at a 1:300 ratio in antibody dilution solution and incubate the cells in the dark either overnight at 4°C or for two hours at RT.
  • For negative controls, incubate the primary HCECs with secondary antibody only (without primary antibodies).
7) Secondary antibody wash: Rinse primary HCECs once using 200–250 µL PBS per well to remove the unbound secondary antibody, followed by three additional washes with 100 µL PBS per well.
8) Mounting and Imaging Preparation:
  • Add 70 µL mounting medium with DAPI (Supplementary Table of Materials) per well to preserve the fluorescence signal and prevent photobleaching.
  • Cover the entire plate with foil and store at 4°C for future use.

#### Confocal Imaging Procedures

High-resolution confocal images are captured using the Opera Phenix Plus high-content imaging system in this protocol (Part No. HH14000000 [Revvity, Waltham, MA, USA]; Supplementary Table of Materials).

1) Select the imaging objective: In this study, we used a 63× (NA: 1.15) water immersion objective to image primary HCECs cultured in 96-well plates.
2) Define FOVs: Ensure that the fields of view (FOVs) are evenly distributed across the well to avoid missing critical areas. For instance, when using a 63× objective, acquire at least 36 FOVs per well arranged in a 6 × 6 grid pattern to ensure comprehensive coverage of the entire well. This approach allows for the acquisition of a sufficient number of FOVs per well from 96-well plates, which are subsequently used for quantifying changes in limbal stem cell numbers in response to increasing Ca^2+^ levels in the culture medium.
3) Capture Z-stack images: To account for variations in protein expression patterns and ensure cells are fully within the focal plane, acquire tile images across a Z-stack range of 10–15 µm. Use intervals of 0.8 µm for the 20x objective (resolution: 0.6 µm/pixel) or 0.5 µm for the 63x objective (resolution: 0.2 µm/pixel).
4) Maintain consistent imaging settings: When comparing antibody expression patterns in primary HCECs cultured in different media (complete growth or differentiation medium), ensure consistent exposure times, exposure power, and Z-stack layer settings across the entire plate. The specific settings for imaging ΔNp63α, Ki67, K3 and DAPI expression pattern in primary HCECs are described in (Table 3).
5) Set excitation/emission channels: Configure the channels based on the fluorophores used for secondary antibodies, such as Alexa-488, Cy3, Cy5, and DAPI (see Supplementary Table of Materials for details).

### Validation of Primary HCECs Function Using Wound Healing Assay

#### In Vitro Wound Healing Assay Protocol

Materials:

• Passage two or three primary HCECs
• Complete growth medium and differentiation medium (See the recipe in the sections on Complete Growth Medium and Differentiation Medium.)
• 10 µL sterile pipette tips
• Sterile 6-well cell culture plates
• Sterile HBSS (without Ca^2+^/Mg^2+^; Supplementary Table of Materials)
• ECHO Revolve Microscope (Model: RVL2-K2) (Any microscope equipped with phase-contrast images is suitable for this assay.)
• 37°C, 5% CO_2_ humidified incubator

Procedures.

1) Cell seeding:
  • Seed the primary HCECs into Matrigel matrix pre-coated 6-well glass bottom microplates at a density of 1.5–3 × 10^5^ cells per well.
  • Allow the cells to grow in a humidified 37°C, 5% CO_2_ incubator until they reach ∼100% confluence.
  • For passage 2 (or later) primary HCECs cultured in NoCa^2+^-added complete growth medium, confluence and well-established cell-cell contacts typically form within two to three days. In contrast, for primary HCECs cultured in the Normal Ca^2+^-added differentiation medium, allow more than three days for a continuous, intact cell-sheet to be observed before initiating the scratch assay.
2) Pre-scratch imaging: Non-scratched Day 0 images of primary HCECs cultured under different conditions are captured using the ECHO Revolve microscope and saved as evidence confirming the formation of a complete monolayer before the scratch assay.
3) Scratch wound creation: A scratch wound is introduced using a sterile micropipette tip to create a straight scratch across the cell monolayer under each culture conditions. Remove the detached cells by gently washing twice with HBSS without Ca^2+^/Mg^2+^ and change into fresh culture different culture medium (“No” or “Normal” Ca^2+^-supplemented growth medium).
4) Post-scratch imaging: Images are taken immediately afterward and set as “Baseline” for subsequent wound healing.
5) Wound-healing monitoring: Wound closure is monitored by microscopic imaging, and changes in the width of the scratch represent the extent of primary HCECs wound healing level under different Ca^2+^-containing medium. Images should be captured at the same region along the scratch at the different time points (e.g., at 24 hours).

### Validation of Primary HCECs Function Using Ca^2+^ Assay

#### In Vitro Ca^2+^ Assay Protocol

Materials.

• Passage 4 primary HCECs, which are cultured with No Ca^2+^-supplemented complete culture medium
• Matrigel matrix pre-coated 96-well black-walled, glass bottom microplates
• Ca^2+^ assay kit (includes assay buffer and probenecid stock solution, 250 mM)
• 1 M HEPES solution
• 1X HBSS solution
• Complete growth medium
• ATP (100 µM)
• Phospholipase C (PLC) inhibitor: U-73122 (10 µM)
• FLUOstar Omega multi-mode microplate reader (or equivalent)
• Pipettes and sterile tips
• 37°C, 5% CO_2_ humidified incubator

Procedure.

##### Preparation of Cells

1) Plate passage 4 primary HCECs into Matrigel matrix pre-coated 96-well black-walled glass bottom microplates at a density of 30,000 cells per well.
2) Allow the cells to grow overnight in a 37°C, 5% CO_2_ humidified incubator until they reach approximately 90% confluence.

##### Preparation of Dye Loading Solution

Prepare the dye loading solution fresh on the day of use.

1) Prepare the assay buffer: The Ca^2+^ assay kit usually includes assay buffer, or you can prepare it by diluting 1 M HEPES with 1× HBSS at a 1:50 ratio.
2) Prepare a 1× dye loading solution: In a sterile container, mix 10 mL of assay buffer with 100 L of the probenecid stock solution gently to ensure homogeneity.
3) Loading the dye:
  • Remove the complete growth medium from the cell cultures.
  • Carefully add 100 µL of the 1× dye loading solution to each well of the 96-well plate. Avoid disturbing the cell monolayer.
  • Incubate the plates in a sterilized 37°C, 5% CO_2_ humidified incubator for 40–45 minutes to allow the dye to load into the cells.

##### Performing the Ca^2+^ Assay

1) Set up the microplate reader: Use a FLUOstar Omega multi-mode microplate reader (or equivalent) with excitation/emission wavelengths set to 494 nm/516 nm. Ensure the reader is equipped with temperature control to maintain the recording environment at 37°C.
2) Plate format and recording:
  • Measure fluorescence changes continuously in each well to capture ATP-induced intracellular Ca^2+^ release.^15^
  • Setup the measurement kinetics as illustrated in Supplementary Figure S1A. Fluorescence signal changes reflecting intracellular Ca^2+^ variations in each well are recorded every two seconds (interval time: 2.00 s). The total recording period is determined by the number of intervals (No. of intervals). The number of flashes (No. of flashes: 120) is set within the middle of the recommended range (0–200) and can be adjusted based on experimental results. The “End time of kinetic window 1” is automatically calculated based on these settings.
  • For ATP, set the injection at 20 seconds using 1 µL at 170 µL/second. The representative ATP-induced intracellular Ca^2+^ release curve is described in Supplementary Figure S1B.
3) Treating the cells with ATP or PLC inhibitor:
  • Continuously record the intracellular Ca^2+^ level and stimulate the primary HCECs with ATP (100 µM, Supplementary Table of Materials) after 20 seconds of baseline recording (the baseline recording time can be adjusted based on the requirements of the specific experiment).
  • Alternatively, pretreat primary HCECs with the PLC inhibitor U-73122 (10 µM, Supplementary Table of Materials) for five minutes before the ATP administration.
  • Set the total recording time per well to five minutes to capture the full trace of intracellular Ca^2+^ changes.
4) Alternative protocol for limited equipment:
  • If a microplate reader is unavailable for simultaneous monitoring, add the dye solution to only two wells per experiment to minimize variations in incubation times.
  • For example, add ATP to the first well and perform the recording. Meanwhile, pretreat the second well with the PLC inhibitor and incubate for five minutes while the first well is being recorded. Add ATP to the second well and repeat the recording.

### Data Analysis of Ca^2+^-Dependent Loss of ΔNp63α Expression in Primary HCECs

ΔNp63α has the highest signal in primary HCECs cultured with no additional Ca^2+^ added to the growth medium compared to other conditions.

1) The brightness, contrast, and threshold settings established under different conditions were consistently applied across all samples to ensure reliable and comparable analysis of ΔNp63α signals. The detailed settings for each channel are described in Table 4.
2) The detailed analysis procedures using ImageJ software are described in Supplementary Figure S2.
3) The percentage of ΔNp63α-labeled cells is quantified by normalizing the number of ΔNp63α-positive cells to the total number of DAPI-stained nuclei in each condition.
4) The percentage of ΔNp63α-positive cells is then plotted against the increasing Ca^2+^ concentrations in the culture medium to illustrate the gradual loss of limbal stem cell properties and the corresponding decrease in ΔNp63α signal intensity in differentiated cells.
5) To compare ΔNp63α expression levels in primary HCECs cultured under different conditions, we performed an ordinary one-way analysis of variance without matching or pairing, followed by multiple comparisons to assess the mean difference between each condition*.* *P* < 0.05 is considered statistically significant.

## Representative Results

**Figure 1.**
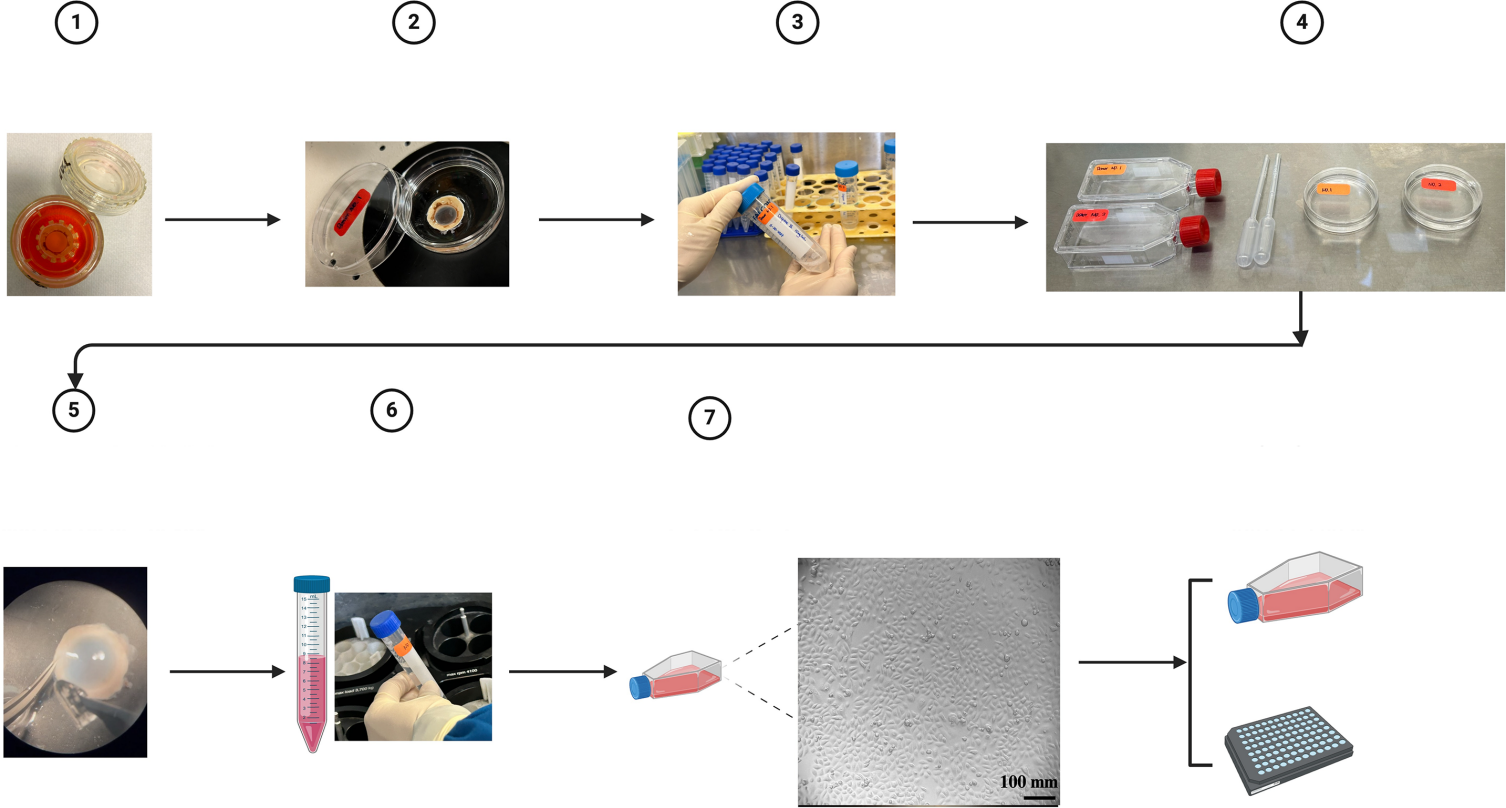
**Experimental protocol of isolating and culturing primary HCECs.** A schematic representation of the protocol for isolating and culturing primary human corneal epithelial cells.

Following the outlined procedures, primary HCECs were successfully isolated from human donor corneoscleral buttons (Fig. 1^2^). The first passage of primary HCECs typically required at least a week to reach 70% to 90% confluence in a T75 flask, with subculturing usually performed on day 6 or 7. From passages 2 to 4, the primary HCECs exhibited a rapid growth rate, with a doubling time of 24–48 hours in 0 mM Ca^2+^-supplemented complete growth medium, making them suitable for high-throughput assays (Fig. 3). By passage 5, primary HCECs usually took three to four days to reach 70% to 90% confluence, compared to the earlier passages, which required only two days. At passage 6, HCECs growth rate significantly declined, and cells could not be passaged further.

**Figure 2.**
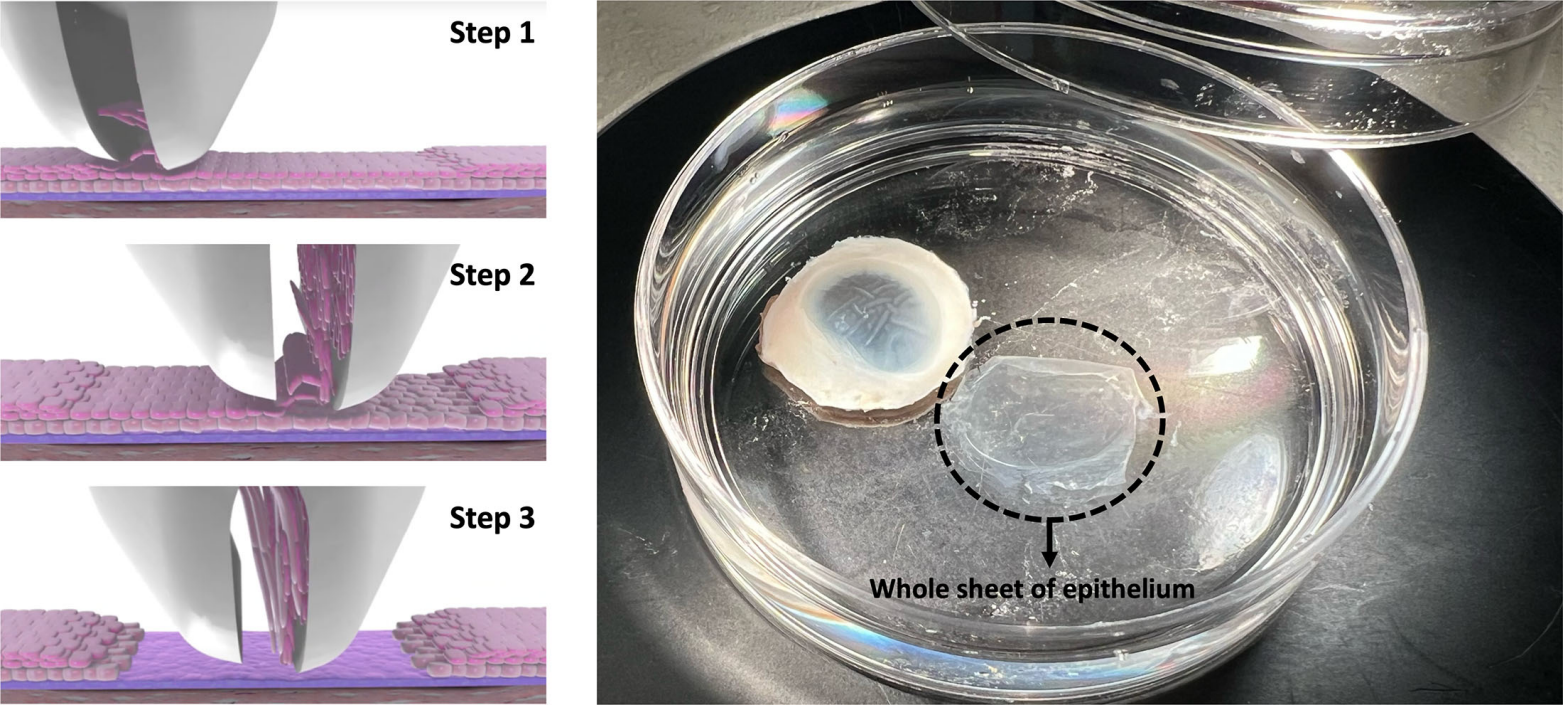
**Isolation of human corneal epithelium.** (**A**) The Epi-Bowman's Keratectomy (EBK) device is positioned in direct contact with the corneal surface (Step 1). As the device moves deeper into the corneal tissue, it selectively lifts and peels the epithelial layer (depicted in *pink*, Step 2). In the final stage of the isolation process, the entire epithelial layer is completely separated from the underlying cornea stroma. (Step 3). (**B**) A petri dish containing the isolated corneal epithelium, immersed in HBSS solution. The entire thin and transparent sheet of epithelium (*circled in black*) indicates successful isolation.

**Figure 3.**
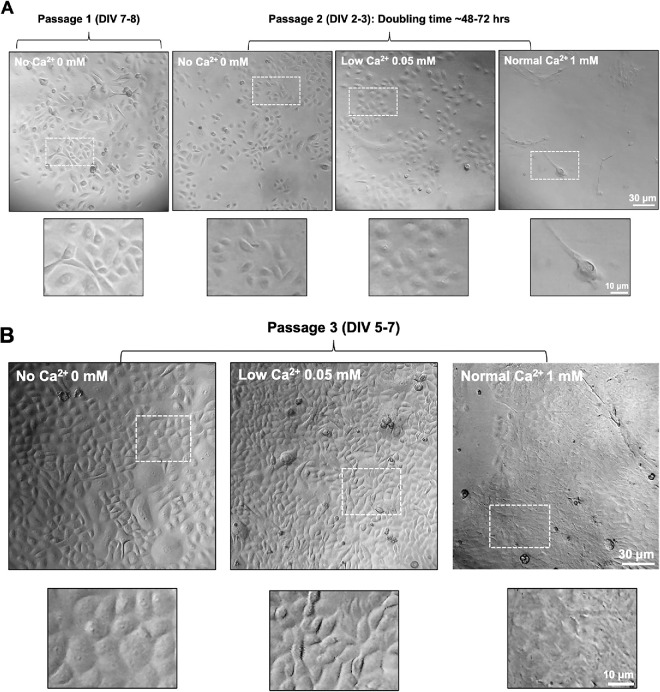
**Morphological changes of primary HCECs in response to Ca^2+^ levels and passage number.** Representative phase-contrast images of primary HCECs cultured at different Ca^2+^ supplementation levels (No = 0, Low = 0.05, and Normal = 1 mM). (**A**) Primary HCECs at passage 1 (DIV 7–8) and at passage 2 (DIV 2–3) cultured in medium with varying Ca^2+^ concentrations (*Top row*). Enlarged views of the boxed regions in the top row highlight individual cell morphology. In the “No” and “Low” Ca^2+^-added complete culture medium, primary HCECs demonstrate a round morphology. However, an elongated morphology is observed in the ‘Normal’ Ca^2+^-containing culture medium (*Bottom row*). (**B**) Passage 3 primary HCECs cultured for a longer period (DIV 5–7) at three culture conditions (*Top row*). Enlarged views of the boxed regions in the top row, detailing individual cell morphology and cell-cell contacts. In the No and Low Ca^2+^-culture medium, primary HCECs show distinct boundaries between cells. However, a sheet-like morphology is present in primary HCECs cultured in ‘Normal’ Ca^2+^- containing medium (*Bottom row*).

### Result 1: Effect of Extracellular Ca^2+^ Concentration on Primary HCEC Differentiation

Previous studies have demonstrated that the composition of culture medium, such as varying Ca^2+^ concentrations, significantly affects cell morphology, differentiation (gene expression), and cell function.^16^ We evaluated the effects of varying Ca^2+^ concentrations on primary HCECs in three groups: No Ca^2+^ (0 mM Ca^2+^- supplemented), Low Ca^2+^ (0.05 mM Ca^2+^-supplemented), and Normal Ca^2+^ (1 mM Ca^2+^-supplemented). Primary HCECs cultured in No Ca^2+^ medium exhibited a robust growth rate from passage 1 onward, reaching 90% confluence rapidly for passage 2 (by DIV2–3, the cells reached approximately 70% confluence). Similarly, cells in low Ca^2+^ medium displayed comparable robust growth. In contrast, primary HCECs cultured in normal Ca^2+^ medium showed slower growth (by DIV5–7 the cells reached approximately 70% confluence) and distinct morphology, characterized by elongated cells and lower cell density (Fig. 3A). Passage 3 HCECs in normal Ca²⁺ medium formed a sheet-like morphology by DIV 5–7 (Fig. 3B). These findings highlight the critical role of Ca^2+^ in regulating primary HCECs growth rate and differentiation.

### Result 2: Expression of Differentiation and Proliferation Markers in Primary HCECs

**Figure 4.**
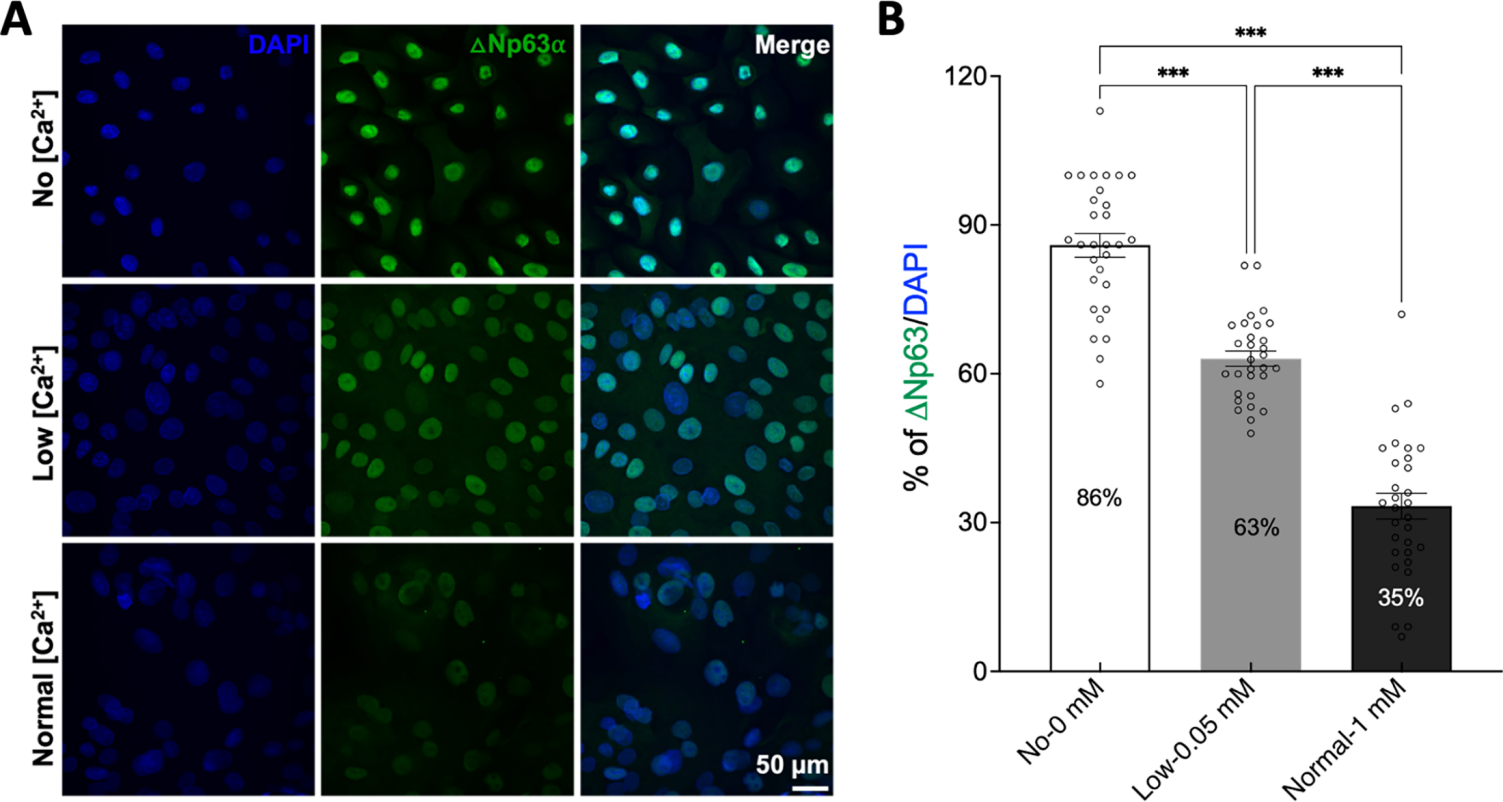
**Effects of Ca^2+^ on the differentiation of primary HCECs.** (**A**) Representative immunofluorescence images of primary HCECs cultured in medium with varying Ca^2+^ concentrations (No, Low, Normal: 0, 0.05, 1 mM Ca^2+^-supplemented). Primary HCECs are stained with DAPI (*left, blue*) and anti-ΔNp63α antibody (*middle*, 1:100, limbal stem cells marker, *green*). The merged images show the co-localization of DAPI and ΔNp63α (*right*, overlay). (**B**) Quantification of ΔNp63α expression using the percentage of ΔNp63α positive cells relative to the total number of cells (DAPI). The graph shows the mean percentage ± SEM from three independent experiments. Statistical significance is determined using one-way ANOVA with Tukey's post-hoc test. ***P < 0.001.

To test whether increased Ca^2+^ concentration influences primary HCECs differentiation status, we used ΔNp63α antibody, one of the most commonly used markers for identifying limbal stem cells (Anti-p40 (ΔNp63α) TP63 antibody, Supplementary Table of Materials).^17^ Our data demonstrated that approximately 86% of the primary HCECs cultured in No Ca^2+^- supplemented complete growth medium were ΔNp63α positive, highlighting the high proportion of limbal stem cell/progenitor cell properties. However, with increasing Ca^2+^ concentrations, primary HCECs gradually lost their stem cell properties, as shown by 63% of ΔNp63α positive cells in low Ca^2+^ and 35% in normal Ca^2+^ conditions (Fig. 4). Our results are consistent with previous findings that ΔNp63α expression is significantly higher in limbal stem cells compared to differentiated corneal epithelial cells.^18^

**Figure 5.**
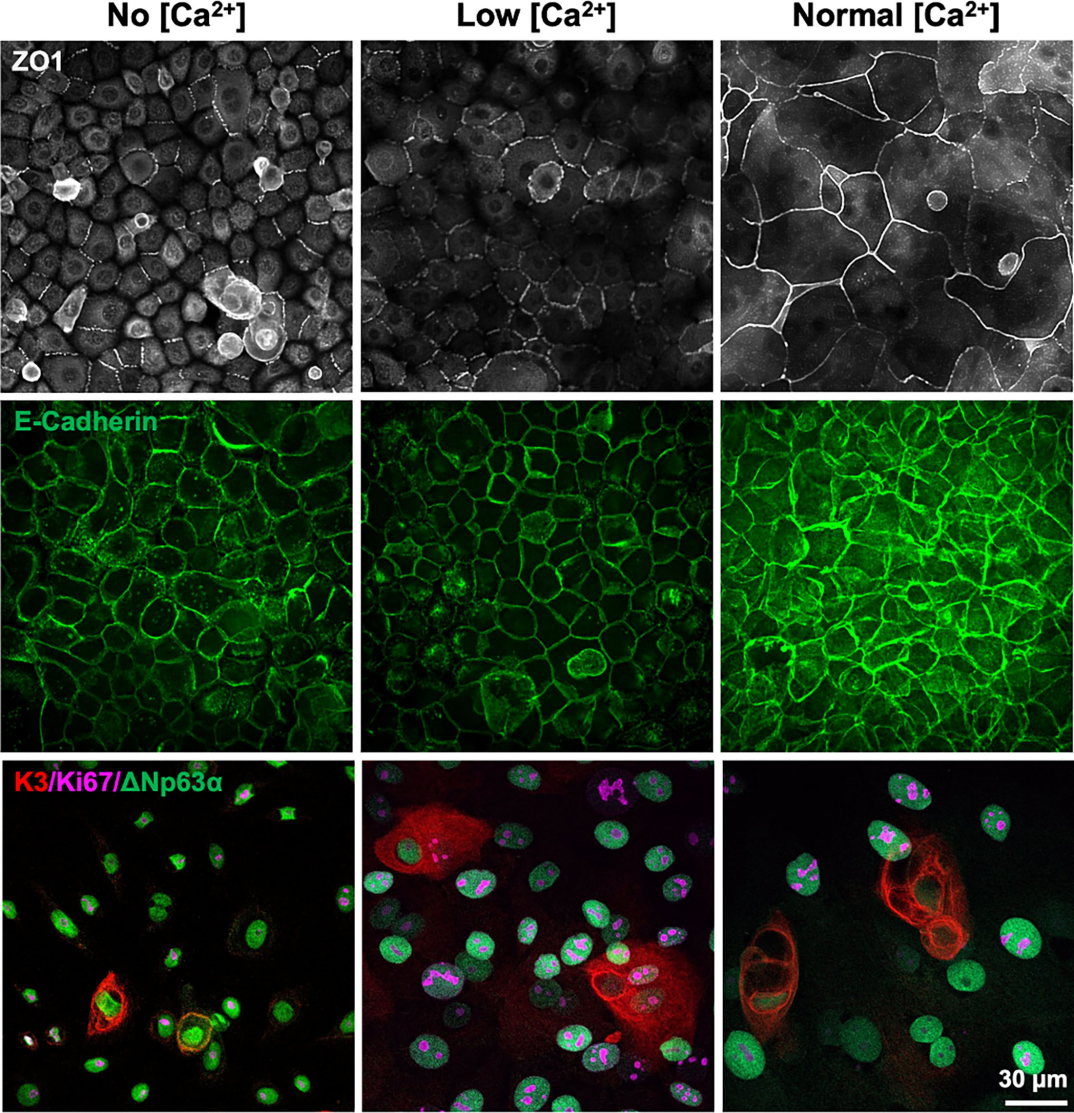
**Characterization of cell marker expression in primary HCECs under different culture conditions.** ZO-1 staining (1:800, *white*) forms a distinct honeycomb pattern at the border of each cell characteristic of partially intact (No and Low [Ca^2+^] conditions) or fully intact (Normal [Ca^2+^] condition) epithelial tight junctions (*First panel*). Immunofluorescence staining shows strong membrane localization of E-cadherin (1:300, *green*) in confluent primary HCECs monolayer cultured in vitro. Under No and Low [Ca^2+^] conditions, E-cadherin forms continuous junctional belts indicative of adherents’ junction integrity, which is restricted to the membrane of adjacent cells with minimal signal in cytoplasm. In contrast, primary HCECs cultured under Normal [Ca^2+^] condition exhibit enhanced E-cadherin expression in both membrane and cytoplasm, suggesting increased junctional assembly and cell-to-cell adhesion (*Middle panel*). Co-staining for K3 (1:300, *red*, corneal epithelial cells marker), Ki67 (1:200, *magenta*, cell cycle indicator), and ΔNp63α (1:100, *green*, limbal stem cell marker). The ΔNp63α limbal stem cell marker is most prominent in the No Ca^2+^ group and is gradually decreased in the Low Ca^2^+ group, showing the weakest signal in the Normal Ca^2+^ group. K3-positive cells are present in all three culture conditions but with larger size correlating with increasing Ca^2+^ concentration. All the K3-positive cells are Ki67-negative across three culture conditions, indicating a lack of proliferative activity in differentiated corneal epithelial cells (*Bottom panel*). Images are acquired using high-content confocal microscopy (Opera Phenix) with 63× water immersion objectives with 1.15 NA.

Additionally, we tested tight junction expression patterns across the three culture conditions using the ZO1 antibody immunofluorescence staining. Under the “No” and “Low” Ca^2+^-supplemented culture medium conditions, there was disruption of tight junctions between HCECs. However, in the primary HCECs cultured in “Normal” Ca^2+^-supplemented medium, there was intact ZO-1 signal indicating well-formed tight junctions clearly delineating the boundaries between neighboring HCECs (Fig. 5, upper panel). The expression of ZO1 is critical for maintaining corneal barrier function and is most prominent in the superficial epithelium compared to the deeper basal epithelium. ZO1 has previously been used as a marker to assess the differentiation level of limbal stem cells^19^ and can also be used to evaluate the differentiation level of primary HCECs in response to varying Ca^2+^ levels. Because E-cadherin is a Ca^2+^-dependent transmembrane glycoprotein, which is crucial for maintaining cell-cell adhesion in epithelial layer,^20^ we further test how Ca^2+^ levels effect E-cadherin expression pattern in primary HCECs using immunofluorescence staining (CD324, Monoclonal antibody [DECMA-1], Supplementary Table of Materials). E-cadherin is primarily located at cell-cell junctions in primary HCECs at all culture conditions, outlining the boundaries of individual cells. Compared to the No and Low Ca^2+^ conditions, primary HCECs cultured under Normal Ca^2+^ exhibit stronger fluorescence intensity, with E-cadherin signals prominently detected at both the plasma membrane and within the cytoplasm (Fig. 5, middle panel).

Last, we confirmed the differentiation status of primary HCECs under the three culturing conditions using the corneal epithelial cell marker, K3 (Polyclonal anti-Keratin K3 antibody; Supplementary Table of Materials), and cell cycle indicator antibody, Ki67 (Anti-Mo/Rt Ki67; Supplementary Table of Materials). The size of K3-positive cells increased in response to higher Ca^2+^ levels in the culture medium. Notably, K3-positive primary HCECs in all three conditions did not coexpress Ki67, indicating the absence of proliferative activity. Because Ki67 is expressed in actively proliferating cells and is absent in quiescent or differentiated cells, these findings suggest that our protocol effectively maintains primary HCECs either as progenitor-like or differentiated-like corneal epithelial cells (Fig. 5, bottom panel).

### Result 3: Effects of Cxtracellular Ca^2+^ on Wound Healing Dynamics in Primary HCECs

**Figure 6.**
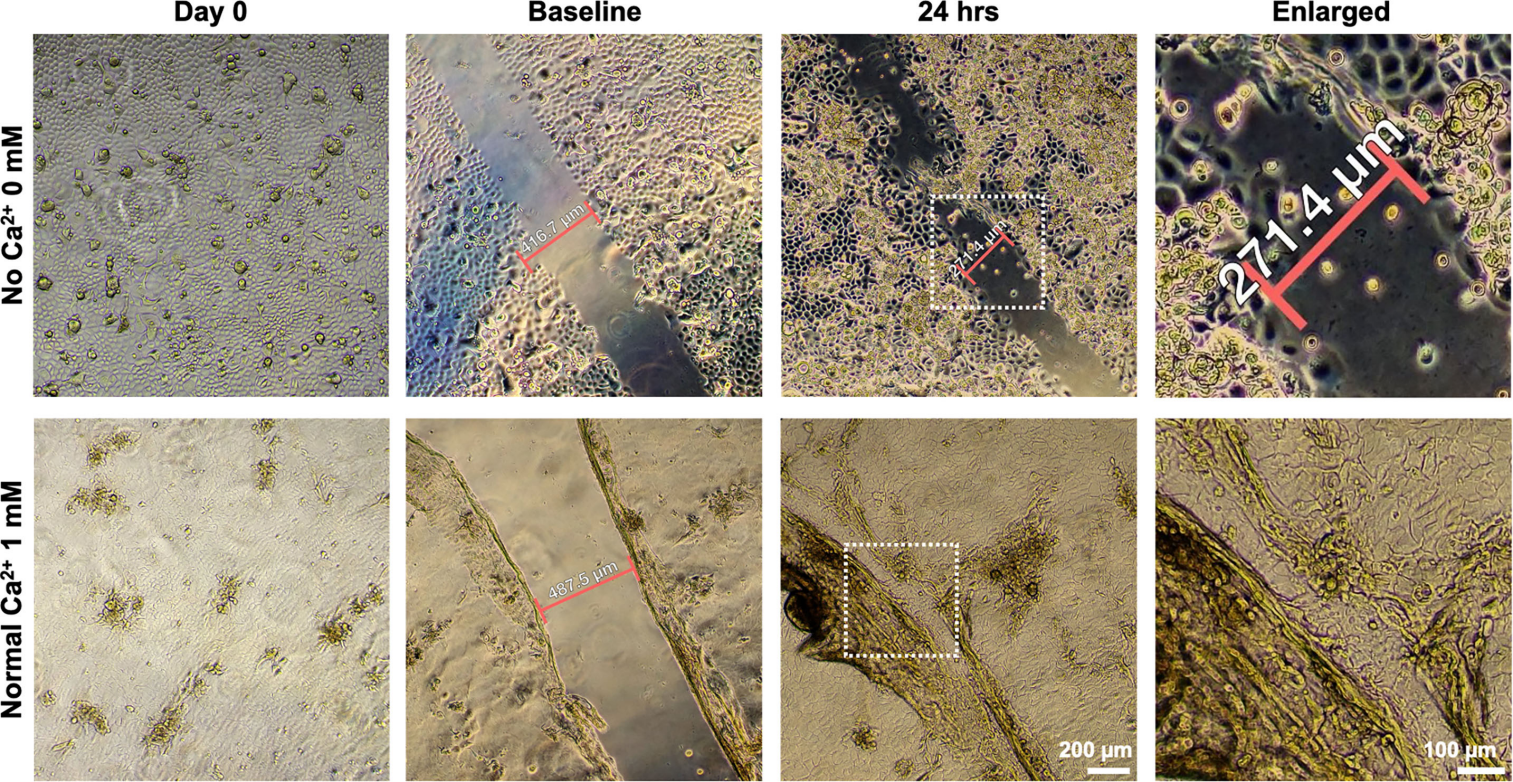
Effect of Ca^2+^ on wound healing in primary HCECs. Representative phase contrast images (4× objective) show scratch wound healing under two different Ca^2+^ conditions: No Ca^2+^-added (*upper panel*) and Normal Ca^2+^-added (*lower panel*) under baseline condition (before scratch) and 24 hours after wounding (after scratch). *Red brackets* indicate the initial or post-24 hours wound widths, and *white dashed boxes* highlight areas of interest where cellular migration and wound closure are evident. Primary HCECs cultured under No Ca^2+^ conditions displayed limited migration leading to less closure. In contrast, primary HCECs cultured in the Normal Ca^2+^ conditions exhibited significant wound closure by 24 hours, represented by the closed gap.

Successful migration and re-epithelialization by primary HCECs confirm that the cultured cells retain dynamic epithelial cells behavior, a key characteristic of cellular maturity and functional integrity.^21^ To further understand how extracellular Ca^2+^ levels influence epithelial cell function, we performed a wound healing assay using primary HCECs cultured in medium supplemented with varying Ca^2+^ concentrations. After generating a defined “Gap” in fully confluent primary HCECs monolayers under both No (Fig. 6, upper panel) and Normal (Fig. 6, lower panel) conditions, we continuously monitored the “Gap” closure over time, assessing the extent of cell migration into the wounded area. Within 24 hours, primary HCECs cultured in Normal Ca^2+^ (1 mM) supplemented culture medium migrated into the wound area and fully closed the gap (Fig. 6, lower panel, “24hr” and “Enlarged”). In contrast, cells cultured in No Ca^2+^ (0 mM) medium exhibit only partially migration, narrowing the gap width but still requiring a longer time to achieve complete closure (Fig. 6, upper panel, “24hr” & “Enlarged”). Our data suggests that the primary HCECs derived from donor corneoscleral buttons retain epithelial cells function and highlighting the essential role of extracellular Ca^2+^ in promoting corneal epithelial repair.

### Result 4: ATP-Induced Ca^2+^ Release in Primary HCECs

**Figure 7.**
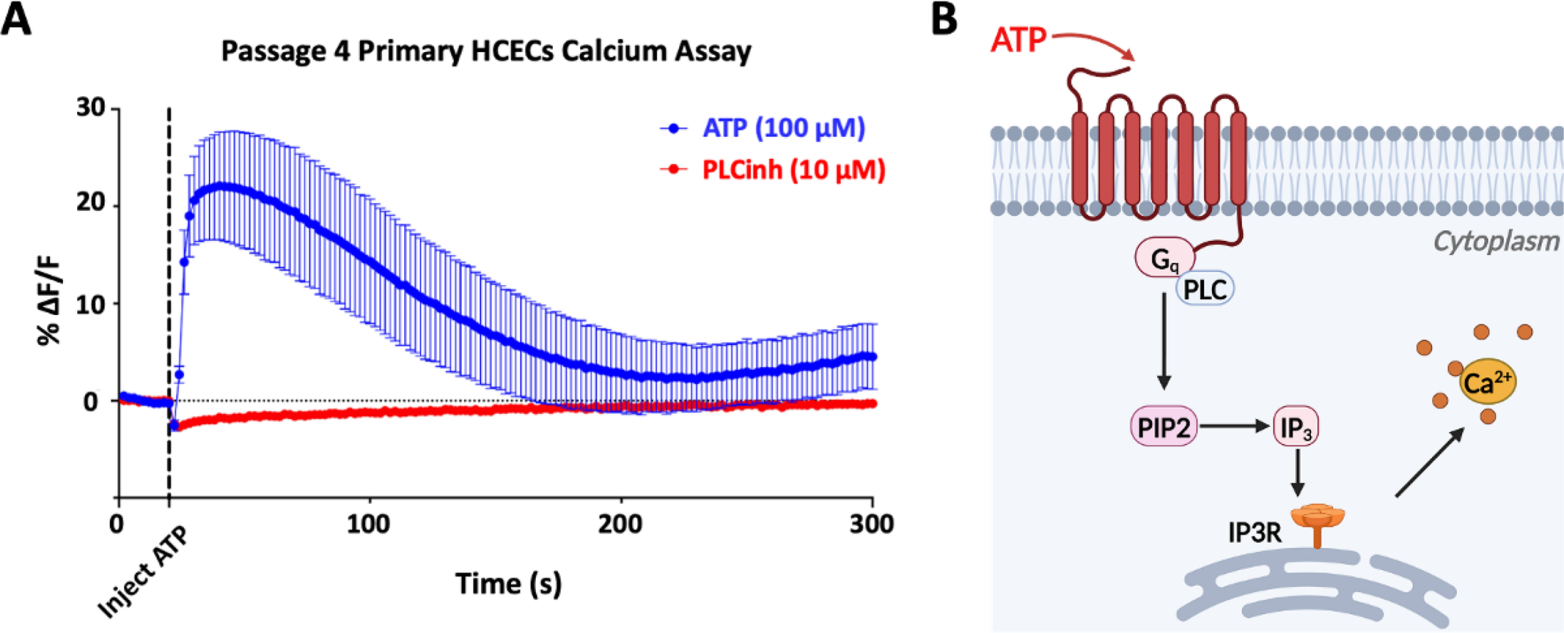
ATP-induced intracellular Ca^2+^ release in primary HCECs. (**A**) Intracellular Ca^2+^ level is measured in primary HCECs. Primary HCECs that are continuously cultured in no Ca^2+^ (0 mM)–supplemented complete growth medium is used for this Ca^2+^ assay. The injection of ATP (100 µM, at 20 seconds) into HCECs immediately induces a transient Ca^2+^ elevation, which gradually returns to baseline over the course of a five-minute recording, as observed through changes in Fluo-4 fluorescence (*blue*). Pretreatment of PLC inhibitor U73122 (10 µM) blocks the ATP-induced Ca^2+^ elevation (*red*). (**B**) Diagram of Ca^2+^ signaling pathway. On ATP binding, the Gq protein is activated and subsequently stimulates PLC, leading to the conversion of PIP2 into IP3, and the release of Ca^2+^ from the endoplasmic reticulum via IP3 receptors.

ATP induces an elevation in intracellular Ca^2+^ concentration ([Ca^2+^]_i_) by binding to purinergic receptors and activating the Gq-PLC signaling pathway in various types of epithelial cells, including the corneal epithelium.^22^^–^^26^ To determine whether the cultured primary HCECs also exhibited a functional response to ATP stimulation, we monitored changes in intracellular Ca^2+^ level using Fluo-4 NW fluorescence. The addition of extracellular ATP (100 µM) induced a transient Ca^2+^ elevation followed by a gradual return to baseline. Pre-treatment of HCECs with the PLC inhibitor U73122 (10 µM) for 5 minutes eliminated the ATP-induced Ca^2+^ elevation (Fig. 7A). These findings suggest that cultured primary HCECs do express functional Gq-coupled purinergic receptors, which mediate binding of external ATP and subsequent intracellular Ca^2+^ elevation through the Gq-PLC signaling pathway (Fig. 7B).

**Table 1.** Corneal Epithelial Cell Growth Kit Components

Component Name	Volume	Final Concentration
Apo-transferrin	0.5 mL	5 µg/mL
Epinephrine	0.5 mL	1.0 µM
Extract P	2 mL	0.4%
Hydrocortisone hemisuccinate	0.5 mL	100 ng/mL
L-Glutamine	15 mL	6 mM
Rh Insulin	0.5 mL	5 µg/mL
CE growth factor	1 mL	Proprietary formulation

**Table 2.** Matrigel Volume Recommendations for Cell Culture

Types of Cell Culture	Volume (Single Unit)
T25 flask	3–5 mL per flask
T75 flask	7–10 mL per flask
96-well plate	50–70 µL per well

**Table 3.** Imaging Parameters for Antibody Expression Analysis in Primary HCECs

		Exposure Setting		Z-Stack (µm)		
Objectives	Channels	Time (ms)	Power (%)	First	Final	Interval (µm)
20× water	DAPI	40	50	−24	1.6	0.8
	ΔNp63α	100	100			
	Ki67	150	100			
	K3	100	100			
63× water	DAPI	40	50	−5.5	4.5	0.5
	ΔNp63α	100	100			
	Ki67	150	100			
	K3	100	100			

**Table 4.** Imaging Settings for ΔNp63α Signal Analysis in Primary HCECs

	No Ca^2+^		Low Ca^2+^		Normal Ca^2+^	
	(0 mM-Supplemented)		(0.05 mM-Supplemented)		(1 mM-Supplemented)	
[Ca^2+^] Channels	ΔNp63α	DAPI	ΔNp63α	DAPI	ΔNp63α	DAPI
Brightness (16 bits)	120	100	120	100	120	100
Contrast (16 bits)	10373	1900	10373	1900	10373	1900
Threshold (Min/Max, 16 bits)	3000/65535	340/65535	3000/65535	262/6535	3000/65535	262/6535
Circularity	0–1.00	0–1.00	0–1.00	0–1.00	0–1.00	0–1.00
Particle size (µm^2^)	70–1000	70–1000	70–2500	70–2500	70–3000	70–3000

## Discussion

This protocol presents an optimized approach for isolating and culturing primary HCECs from donor corneoscleral buttons, addressing key challenges in generating high-purity and viable cell populations to study the ocular surface. Critical steps include the precise isolation of the corneal epithelial layer using Dispase II digestion and a specialized surgical device, ensuring that HCECs are separated with minimal stromal contamination. The successful culture of primary HCECs across several passages relies heavily on the optimization of culture conditions, particularly the Ca^2+^ concentration, which plays a pivotal role in regulating cellular morphology and differentiation status. In our previous studies, we cultured the immortalized HCECs in commercial Ca^2+^-free medium (Keratinocyte SFM without CaCl_2_, Catalog No. 37010-022; Gibco, Thermo Fisher Scientific, Waltham, MA, USA), and these in vitro cultured immortalized HCECs maintained their ability of responding to the external lysophosphatidic acid receptor (LPAR) agonist stimulation, exhibiting an elevated intracellular Ca^2+^ level.^27^ In this study, we cultured primary HCECs in a very low Ca^2+^-containing complete culture medium (0.0612 mM) without additional Ca^2+^ supplementation, those primary HCECs keep their limbal stem cell-like property, with their functionality further validated using a Ca^2+^ assay (Fig. 5A). By adjusting media Ca^2+^ levels, this protocol not only ensures effective cell growth and proliferation but also promotes HCECs’ differentiation, mimicking the natural behavior of these cells in vivo. We also described the expression pattern of calcium-sensing receptors (CaSR) and their role in regulating ocular surface hydrarion.^28^ In addition to inducing the epithelial cells’ differentiation, variations in Ca^2+^ levels may represent a potential therapeutic approach for treating ocular disease.

Modifications to the method, such as adjusting the Ca^2+^ concentrations in the culture medium, can mitigate issues like slow cell growth or morphological changes. A key troubleshooting step that sometimes helps us to acquire a higher quantity of primary HCECs is extending the digestion time, which can range from 16 to 24 hours at 4°C. Also, ensuring high-quality donor corneal tissues can improve yield. The use of freshly made Matrigel matrix solution to coat the culture surface supports cell attachment and survival, helping maintain the native cell morphology during culture.

Despite its advantages, this method of culturing primary HCECs has limitations. The variability in the quality and yield of primary HCECs can arise from donor corneal tissue conditions, which can impact reproducibility. Additionally, while the protocol allows for sub-culturing HCECs up to five passages, the cells’ viability and growth rate decrease with each subsequent passage, limiting the duration of experiments. Moreover, although the protocol is designed for two-dimensional cell culture in vitro applications, further adaptations for three-dimensional cell culture may better mimic in vivo studies.

This method offers notable improvements over alternative techniques, such as immortalized corneal epithelial cell lines, which may not fully replicate the authentic properties of primary human cells. By providing a more accurate model of corneal function and differentiation, this protocol highlights how the extracellular Ca^2+^ level can be adjusted to fit different scientific questions by targeting the limbal-stem cells (No Ca^2+^-added) or differentiated corneal epithelial cells (Normal Ca^2+^-added).

Our protocol improves the predictive value of preclinical studies and facilitates the evaluation of potential therapeutic agents. Beyond corneal studies, this protocol can also be adapted for research on other epithelial cell types, broadening its applications in cell biology and drug discovery.

## Supplementary Material

Supplement 1

Supplement 2

Supplement 3

Supplement 4
