# Supplementary material for: Optimized Protocol for Isolation and Culture of Primary Human Corneal Epithelial Cells
Source: Transl Vis Sci Technol. 2025 Oct 22;14(10):28. doi: 10.1167/tvst.14.10.28 (PMC12553468; doi:10.1167/tvst.14.10.28)
Supplement: Supplement 1 [file tvst-14-10-28_s001.docx]

**
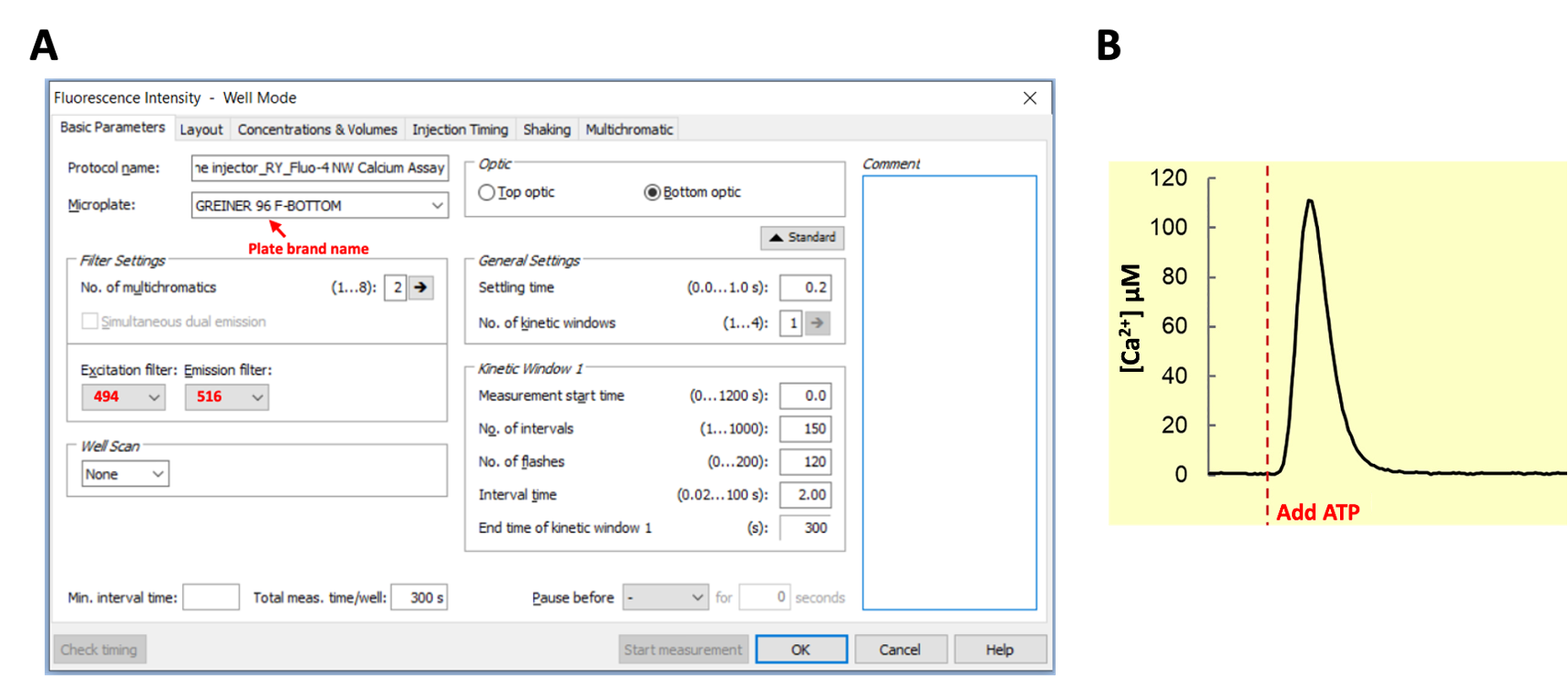
**

**Supplementary Figure 1. A.** Plate format setup for the Ca^2+^ assay. **B.** Representative trace showing the time-dependent changes in intracellular Ca^2+^ induced by ATP.

**
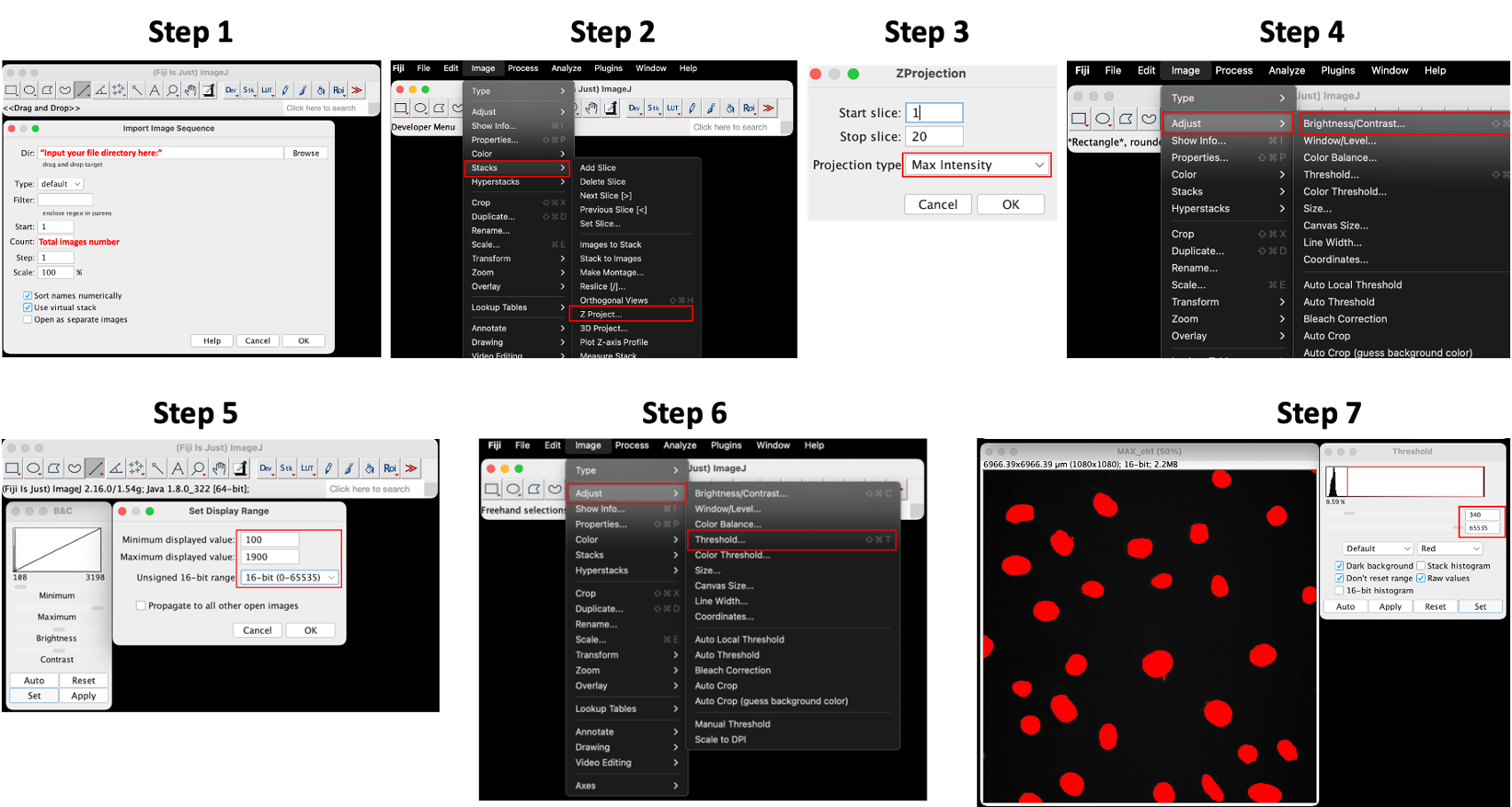
**

**
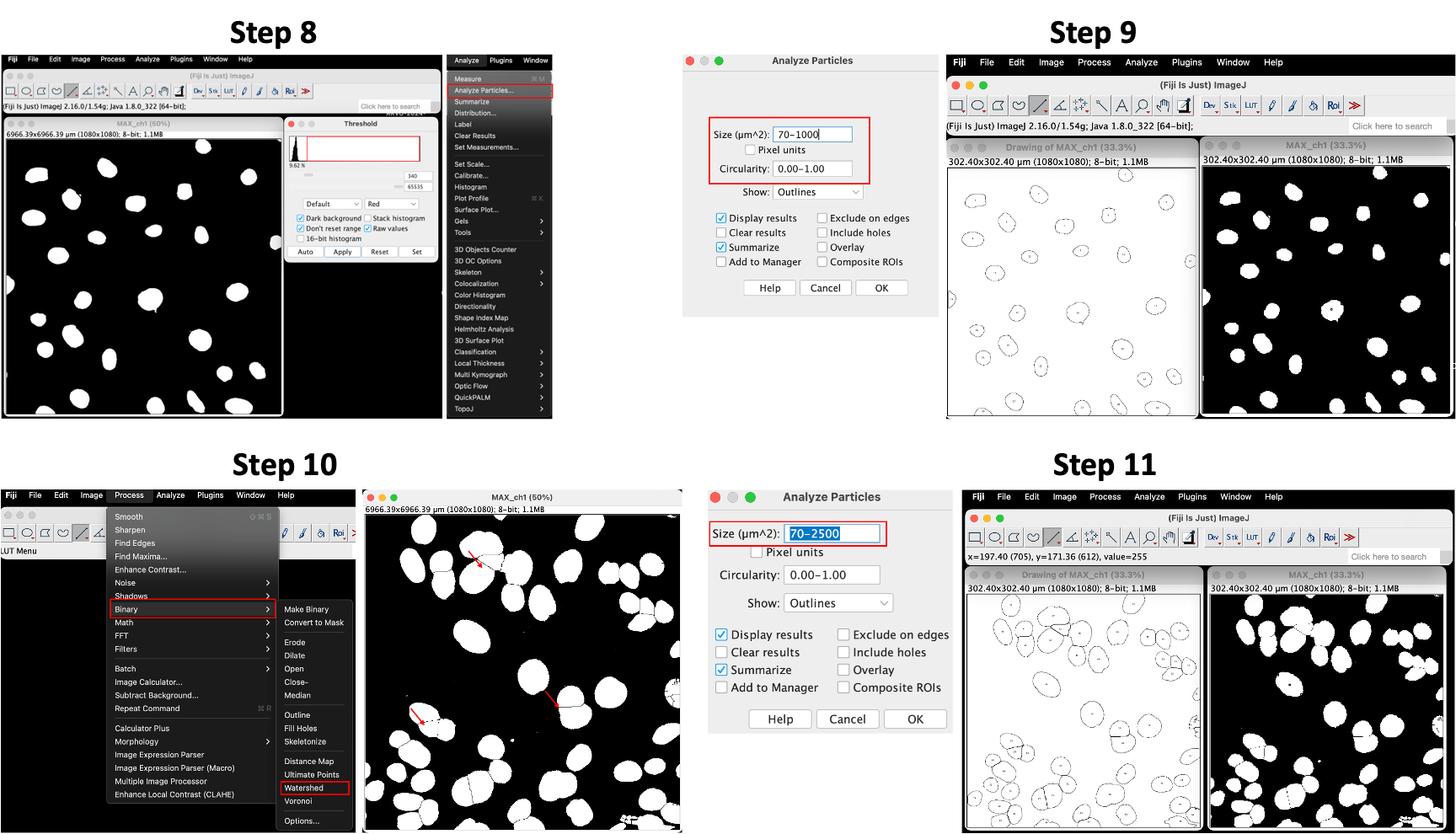
**

**Supplementary Figure 2 (Steps 1-11).** The detailed steps for analyzing the number of ΔNp63-positive cells per condition, as well as calculating the total number of cells based on the DAPI signal, using Fiji (ImageJ distribution) software^26^.
